# Subversion of Autophagy in Adherent Invasive *Escherichia coli*-Infected Neutrophils Induces Inflammation and Cell Death

**DOI:** 10.1371/journal.pone.0051727

**Published:** 2012-12-14

**Authors:** Abderrahman Chargui, Annabelle Cesaro, Sanda Mimouna, Mohamed Fareh, Patrick Brest, Philippe Naquet, Arlette Darfeuille-Michaud, Xavier Hébuterne, Baharia Mograbi, Valérie Vouret-Craviari, Paul Hofman

**Affiliations:** 1 Institute for Research on Cancer and Aging, Nice, Institut National de la Santé et de la Recherche Médicale U1081/Centre National de la Recherche Scientifique UMR7284, Team 3, Nice, France; 2 Université de Nice-Sophia Antipolis, Faculté de Médecine, Nice, France; 3 Equipe Labellisée Fondation Association pour Recherche sur le Cancer, Villejuif, France; 4 Institut National de la Santé et de la Recherche Médicale U898, Nice, France; 5 Centre d’imunologie de Marseille-Luminy, UMR 631, Marseille, France; 6 Institut National de la Santé et de la Recherche Médicale U1071, Clermont-Ferrand, France; 7 Institut National de la Recherche Agronomique, USC2018, Clermont-Ferrand, France; 8 Département de Gastroentérologie, Hôpital de l’Archet, Centre Hospitalier Universitaire de Nice, Nice, France; 9 Laboratory of Clinical and Experimental Pathology and Human Biobank, Centre Hospitalier Universitaire de Nice, Pasteur Hospital, Human Biobank, Nice, France; Monash University, Australia

## Abstract

Invading bacteria are recognized, captured and killed by a specialized form of autophagy, called xenophagy. Recently, defects in xenophagy in Crohn’s disease (CD) have been implicated in the pathogenesis of human chronic inflammatory diseases of uncertain etiology of the gastrointestinal tract. We show here that pathogenic adherent-invasive *Escherichia coli* (AIEC) isolated from CD patients are able to adhere and invade neutrophils, which represent the first line of defense against bacteria. Of particular interest, AIEC infection of neutrophil-like PLB-985 cells blocked autophagy at the autolysosomal step, which allowed intracellular survival of bacteria and exacerbated interleukin-8 (IL-8) production. Interestingly, this block in autophagy correlated with the induction of autophagic cell death. Likewise, stimulation of autophagy by nutrient starvation or rapamycin treatment reduced intracellular AIEC survival and IL-8 production. Finally, treatment with an inhibitor of autophagy decreased cell death of AIEC-infected neutrophil-like PLB-985 cells. In conclusion, excessive autophagy in AIEC infection triggered cell death of neutrophils.

## Introduction

Host survival is dependent on effective recognition and killing of pathogens. Professional phagocytes such as neutrophils, macrophages and dendritic cells directly kill microorganisms through phagocytosis and via the production of reactive oxygen intermediates, proteolytic enzymes and cytokines. Among these cells, neutrophils are the first line of innate defence recruited at the site of infection. Upon pathogen clearance, the rapid execution of neutrophil death is central for the resolution of infection. These cells have a short life span in the circulation–only 5 days–and rapidly go into apoptosis [1]. Conversely at sites of inflammation, the lifespan of neutrophils is dramatically prolonged and other forms of cell death than apoptosis have been described; including NETosis, a programmed cell death that leads to neutrophil extracellular trap formation [2], and autophagy [3], [4], [5]. However, the type of neutrophil cell death induced by bacterial pathogens still remains elusive. This issue is critical as excessive or dysregulated neutrophil responses contribute to persisting tissue damage that underlies many inflammatory diseases.

Adherent-invasive *Escherichia coli* (AIEC) have been isolated from the intestinal mucosa of patients with Crohn’s disease (CD), a chronic inflammatory bowel disease of uncertain pathogenesis. The AIEC reference strain LF82 is able to adhere to intestinal epithelial cells [6], to invade epithelial cells, and to survive and replicate within macrophages [7], [8]. Following uptake, AIEC are wrapped into a host-cell derived phagosome, which matures from an early to late phagosome and fuses with lysosomes to form the degradative phagolysosome [9]. The harsh environment of phagolysosomes finally destroys the microorganism. However, AIEC pathogenic bacteria replicate in high numbers in mucosal cells of CD patients [10]. Recently, xenophagy, a specialized form of autophagy, has emerged as a critical mechanism in the capture and lysis of invasive bacteria [11], [12], [13]. Moreover, most studies showing xenophagy-mediated killing of bacteria concern monocytes [14], macrophages [15], [16], or epithelial cells (for review see ref [17]).

The role of xenophagy in human neutrophils, both in bacterial killing and in the execution of neutrophil cell death, has not been elucidated to date. To address this issue, we focused our attention on autophagy induced by the interaction between neutrophils and the AIEC LF82 strain.

## Materials and Methods

### Cell Culture

Venous blood was collected from healthy donors after obtaining informed consent. Neutrophils were then isolated from whole blood using a gelatin-sedimentation technique as previously described in [18]. Blood neutrophils are extremely short-lived cells that are not transfectable. To get around this limitation, we used the human myeloid cell line, PLB-985, that differentiates *in vitro* into mature neutrophil-like cells [19] and have been described to produce ROS on challenge with *E. coli* [20].

PLB-985 cells (a gift from Pr Sylvie Chollet-Martin, Paris, France) were maintained in RPMI 1640-GlutaMax medium (Gibco, Invitrogen, France) supplemented with 10% fetal calf serum (BioWhittake Lonza; Verviers, Belgium), 50 U/ml penicillin, and 50 µg/ml streptomycin at a density of 0.2–1×10^6^ cells/ml. For neutrophil-like differentiation, exponentially growing cells (0.2×10^5^/ml) were cultured in the presence of 0.5% *N, N*-dimethyl formamide (DMF; 08796DM, Sigma), 1% Nutridoma-SP (11011375001, Roche; Mannheim, Germany), and 0.5% FCS, as previously described [20], [21].

To inhibit autophagy we engineered a cell line silenced for ATG5 expression. Short hairpin RNA (shRNA) lentivirus against *atg5* (Sigma, human NM_004849) and control shRNA lentivirus (SHC002V, Sigma) were transduced into PLB-985 cells. *Atg5* shRNA-transduced cells were selected in puromycin and assayed for *Atg5* silencing by qRT-PCR using specific primers (see Table S1 for sequences).

### Bacterial Strains

The AIEC strain LF82 was isolated from a chronic ileal lesion of a patient with Crohn’s disease [22]. We used the K12 *E. coli* strain C600 as a non-invasive and non-pathogenic control bacteria. Bacteria were cultured as previously described [22].

### AIEC Infection, Invasion and Survival Assay in Human Neutrophils and Differentiated PLB-985 Cells

Prior infection of neutrophils and differentiated PLB-985 cells (1×10^6^ cells/condition) were incubated 30 min in RPMI supplemented with 1% FCS. Cells were then incubated with bacteria at a multiplicity of infection (MOI) of 50. Intracellular survival was assessed by the gentamycin assay as described in [7]. All infections were performed in duplicate, and each experiment was repeated 3 times.

### Analysis of Autophagy

As recommended in [12], we used transmission electron microscopy (TEM), confocal microscopy and immunoblotting to study the consequences of AIEC infection on autophagy in neutrophils. The formation of autophagic vesicles was analysed at the ultrastructural level using a Jeol EXII transmission electron microscope. Human neutrophils or differentiated PLB-985 cells were infected for the indicated time and fixed with ice-cold 3% glutaraldehyde in 0.1 M Na cacodylate, pH 7.4 for 2 h. Fixed cells were processed as described in [2].

To confirm the activation of autophagy we studied the formation of autophagolysosome by indirect immunofluorescence staining and confocal microscopy. In particular, the subcellular distribution of LC3-II, a specific marker of autophagosomes, (1∶500, mouse, clone 5F10, Nanotools) and LAMP-1, the lysosome-associated membrane protein-1, (1∶1000, goat, Santa Cruz biotechnology) were analysed.

Finally, to confirm autophagy, we studied the autophagic flux. For that purpose control- or infected-cells were incubated with specific lysosomal protease inhibitors (Pepstatin A and E64d, 10 µg/mL, Sigma) or the inhibitor of autophagy 3-methyladenine (3-MA, 5 mM, Sigma) and the levels of the autophagosome-associated LC3-II protein (Nanotools, clone 5F10, dil. 1/1000, which preferentially recognizes LC3-II), and the substrate p62/SQSTM1 protein (BD Transduction Laboratories™; # 610833, dil. 1/1000) were analyzed by immunoblotting.

To inhibit autophagy, cells were treated with 3-methyladenine (3-MA, 5 mM, Sigma), which inhibits the formation of autophagic vesicles as described in [12].

### RT-qPCR Procedure

Total RNA was isolated from cells with TRIzol Reagent (Invitrogen) and 1 microgram was reversed transcribed using the SuperScript III First Strand kit (Invitrogen). Quantitative RT-PCR analysis was performed on a 7500 Real-Time PCR system and SYBR® Green PCR Master Mix (Applied Biosystems; Life Technology, California, USA) as previously described in [1]. Relative changes in gene expression (see Table S1 for primer sequences) are reported as fold changes compared to the untreated cell sample.

### Detection of Neutrophil Cell Death

To study cell death induced by intracellular bacteria, cells were infected at a MOI of 50 for 1 h. rinsed in RPMI containing 0.5% FCS and gentamycin (100 µg/ml) and incubated in the same medium for 5 h. When indicated, a pan caspase inhibitor (z-VAD FMK (Bachem, N-1560; 100 µM) or an inhibitor of autophagy (3-MA, 5 mM) was added to cultures 30 min before infection. Infected cells were washed in PBS and the features of dead cells were measured by uptake of propidium iodide and activity of caspase 3. For propidium iodide uptake, infected cells were stained for 5 min with the red-fluorescent probe propidium iodide (5 µg/mL) and the acquired fluorescence within 30 min was measured with a FACS Calibure flow cytometer. Analyses were performed using CellQuest software (BD Biosciences). For caspase 3 activity, lysates (50 µg) were incubated with the caspase-3 fluorogenic substrate Ac-DEVD-AMC (0.2 mM, ALEXIS Biochemicals). Hydrolysis was followed at different times at 37°C as described by the manufacturer. The results were expressed as arbitrary units. For positive controls, cells were incubated with etoposide phosphate (100 µg/ml; Sigma) or staurosporine (10 µM; Sigma).

Alternatively, procaspase-3 cleavage (a signature of cells undergoing apoptosis) was analyzed by immunoblotting using an anti-caspase-3 antibody (1∶1000, rabbit, Cell Signaling Technology). In addition, the cleaved fragment of poly (ADP-ribose) polymerase (PARP, 85 kDa), a caspase-3 substrate, was analyzed with anti-PARP (1∶1000, rabbit, Cell Signaling Technology).

### Statistical Analysis

Results are expressed as means ± sem. PLB-treated and untreated cells were compared using the Student *t* test. The differences between AIEC LF82 and K12 infection were compared using the Kruskall Wallis test followed by the Mann Whitney U test. A *P* value less than 0.05 were considered significant.

## Results

### Intracellular Survival of AIEC within Neutrophils

**Figure 1:**
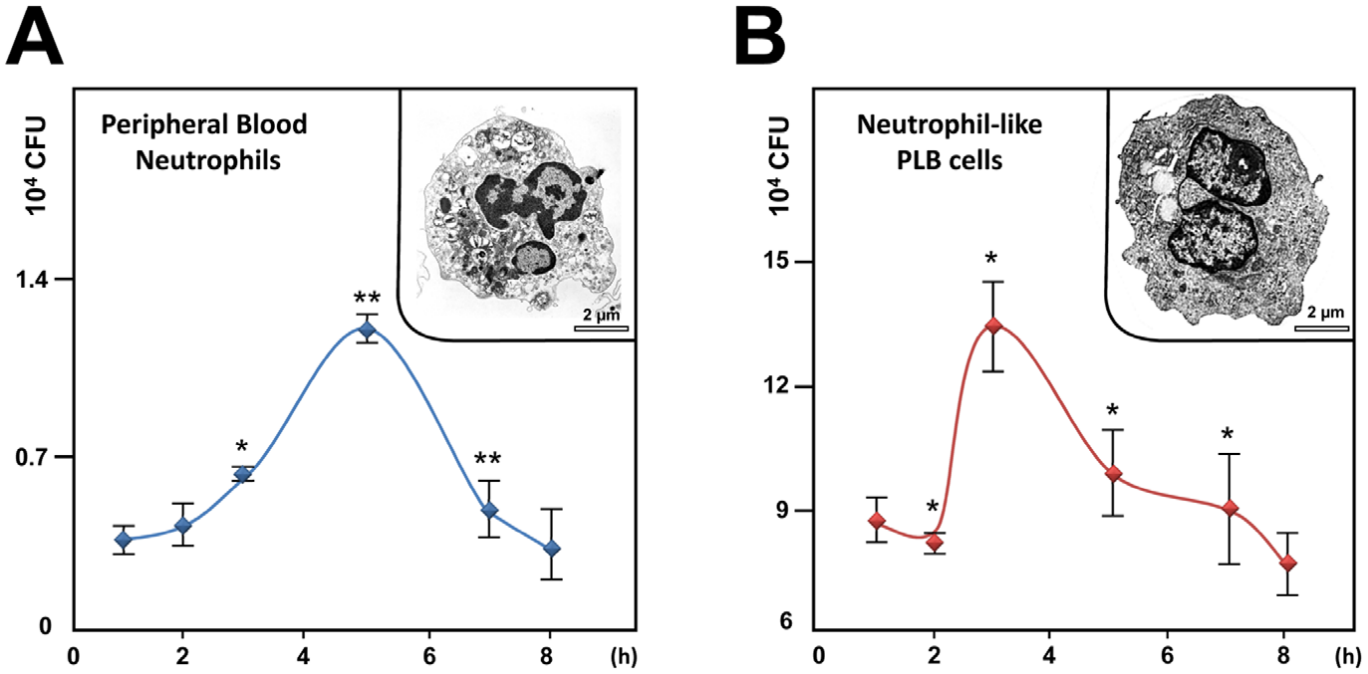
AIEC invade and replicate within PMN. A) Peripheral blood PMNs or B) differentiated PLB-985 cells were infected with AIEC LF82 at a multiplicity of infection (MOI) of 50. The survival of bacteria was measured by the gentamicin protection assay. After 1 h of infection (50 MOI), peripheral blood PMNs and differentiated PLB-985 cells were incubated with gentamicin (100 µg/ml). At the indicated times, cells were washed with PBS and lysed with PBS 1% Triton X-100. The CFUs were determined on LB agar plates. The data are representative of 5 independent experiments. Inset: representative transmission electron micrograph (TEM) pictures of differentiated PLB-985 cells showing the characteristic segmented nuclei of mature PMN with connected lobes. * p<0.05, ** p<0.02.

Since AIEC are able to adhere to and invade epithelial cells and macrophages [7], [8], the aim of this study was first to evaluate whether AIEC LF82 invade and survive within neutrophils and second to analyze the consequence of survival of intracellular bacteria on neutrophil life span. For this purpose, we first infected peripheral blood neutrophils with AIEC LF82 at a MOI of 50 and followed survival of bacteria over time in presence of gentamicin. Recovery of intracellular bacteria showed that live bacteria invade neutrophils. Interestingly, AIEC LF82 bacteria were able to replicate, as a 3-fold increase in the intracellular counts of AIEC was observed 5 h post gentamicin treatment (Fig. 1A). To study in depth the consequences of AIEC infection in neutrophils, we used the human myeloid cell line PLB-985 differentiated into neutrophils [19], [21]. As illustrated in the insets of Fig. 1, these differentiated cells were morphologically similar to human neutrophils with a multilobed and fragmented nucleus. Further, differentiation was confirmed by: *i*) morphology of May-Grünwald-Giemsa stained cells and *ii*) cell surface expression of CD11b (see Fig. S1). When neutrophil-like differentiated PLB-985 cells (Fig. 1B) were infected with AIEC LF82 bacteria, we observed that bacteria survived intracellularly and were able to divide with a maximum observed 3 h post gentamycin treatment. In both cell types, AIEC replication was transient, as the CFU returned to the basal levels 8 h after gentamycin treatment. Therefore, we wondered whether this rapid eradication of the pathogen could result from effective intracellular killing of bacteria and/or increased neutrophil cell death.

### AIEC LF82 Subverts the Autophagic Pathway at the Degradation Step to Promote their Survival

Autophagy can play an important part in protecting host cells during bacterial infection and several pathogens have developed strategies to evade or even exploit this pathway [23]. Activation of autophagy was examined by formation of microtubule-associated protein 1 light chain 3-II (LC3-II), a specific and sensitive marker of autophagy [12].

**Figure 2:**
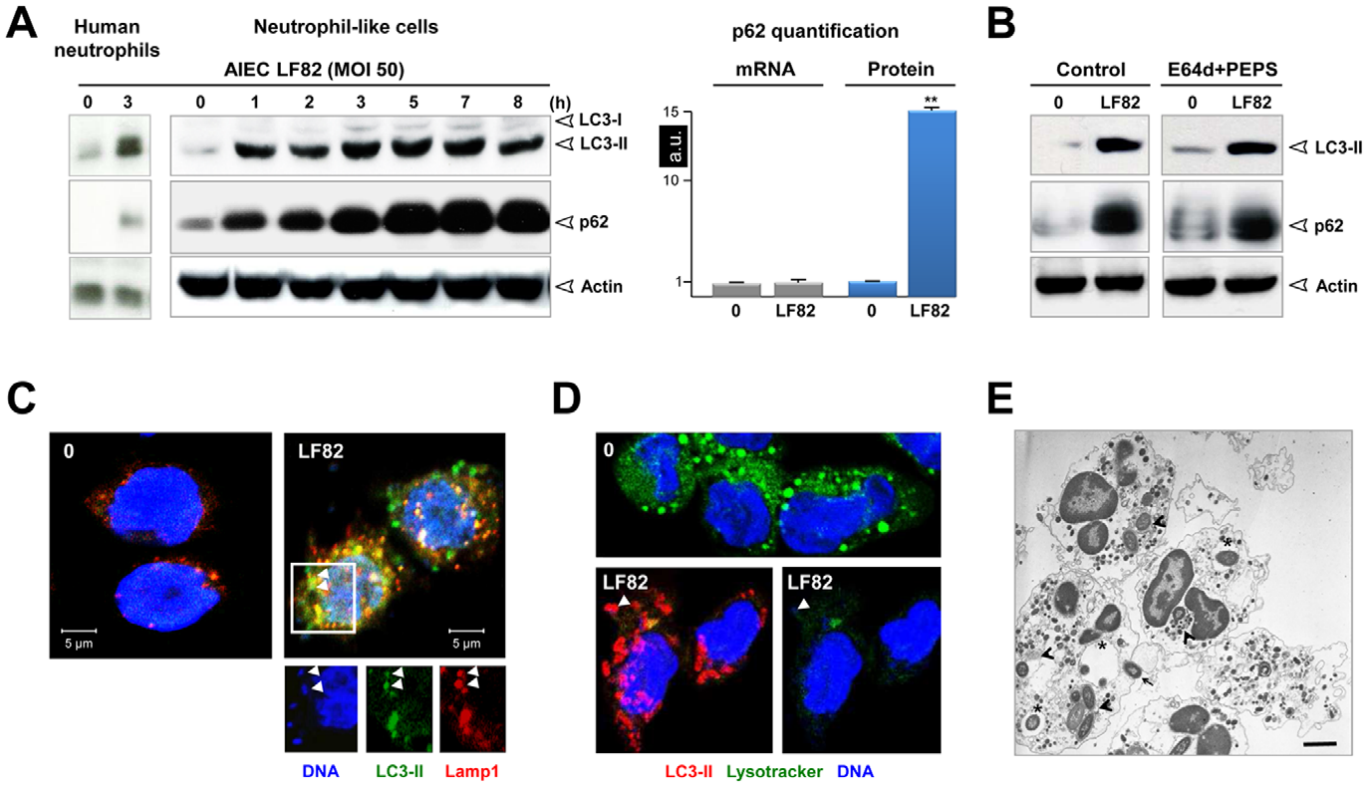
Inhibition of autophagic flux by AIEC LF82 infection. Human neutrophils or neutrophil-like PLB-985 cells were infected with AIEC LF82 at a MOI of 50 for 1 h than gentamicin (100 µg/ml) was added for 3 h. Cells were and processed for immunoblotting (**A,** left panel, **B**), quantitative RT-PCR (**A** rigth panel), immunofluorescence (**C**, **D**) and ultrastructural TEM analysis (**E**). (**A**) Time-dependent accumulation (1–8 h) of LC3-II and p62 in infected human neutrophils or neutrophil-like PLB-985 cells compared to uninfected cells analyzed at the end time point. Longer exposure detects the LC3-I band. We checked that AIEC infection did not affect p62 mRNA levels by qRT-PCR analysis (**A** right panel). Data are means ± SEM of three experiments. ** p<0.001. (**B**) Autophagic flux was analysed by immunoblot analysis in differentiated PLB-985 cells infected for 3 h with AIEC LF82 bacteria (MOI 50) in the absence or in the presence of E64d/PEPS. Actin was used as a loading control. Control unstimulated cells were analysed at the end time point. (**C**) Representative confocal images of control (0) or infected cells (LF82) (3 h post infection) showed the colocalization of bacteria with LC3-II and LAMP-1 proteins as indicated by yellow punctiform staining. Insets highlight individual staining of bacteria (DNA staining, blue), LC3-II (Alexa 488, green) and LAMP-1 (Alexa 594, red). (**D**) Representative confocal micrographs of control (0) and LF82 infected cells (LF82) showing the co-localization of bacteria (DNA staining, blue) within autophagic (LC3-II positive, Alexa 594, red) but not acidic compartments (LysoTracker negative, green). (**E**) Representative TEM images showing bacteria within endosomes (asterisk), autophagosomes (arrowheads) or free in the cytosol (arrow) in LF82-infected cells (3 h post infection). Bar = 2 µm.

Following infection with the AIEC LF82 strain, formation of LC3-II was strongly induced in human neutrophils, demonstrating for the first time that live intracellular *E. coli* are efficient inducers of autophagy in this cell type (Fig. 2A, left panel). A study of the kinetics of infection of differentiated neutrophil-like PLB-985 cells revealed that the band corresponding to LC3-II appeared in a time-dependent manner. LC3-II formation started at 1 h post AIEC infection to reach a plateau by 3 h, which lasted 7 h before slowly declining at 8 h. Physiologically, autophagy is a dynamic process and autophagosomes are formed and degraded within seven minutes. As a result, only a few autophagic vesicles and low LC3-II levels are detected under nutrient starvation [24]. While accumulation of autophagosomes has been documented in AIEC-infected epithelial cells (HeLa, Hep-2 and Intestine-407) [6], no information is available regarding the autophagic flux in AIEC-infected cells.

Sustained accumulation of LC3-II in differentiated neutrophil-like cells may reflect either increased formation or impaired degradation of autophagic vesicles [12]. To discriminate between these two distinct scenarios, we assessed the accumulation of LC3-II in the presence of the lysosomal protease inhibitors E64d and Pepstatin. Cells treated with E64d/Pepstatin and infected with AIEC LF82 did not exhibit further accumulation of LC3-II compared to infected cells (Fig. 2B). Likewise, the autophagic substrate, p62/SQTM1 [25], rapidly accumulated in AIEC-infected cells and was unchanged when autophagy was impaired by E64d/pepstatin treatment, which further supported inhibition of the autophagic flux (Fig. 2A–B). The increase in p62 protein levels in AIEC-infected cells corresponded to a reduction in the autophagic flux as comparable levels of *p62* mRNA were measured in control and infected cells (Fig. 2A, right panel). The same p62 accumulation was observed in AIEC-infected human neutrophils. Together, this suggests that AIEC infection impaired the autophagic flux in neutrophils.

We then questioned which step in the autophagy pathway was stopped/delayed by AIEC infection. Autophagy begins with the formation of a double-membrane autophagosome that stains positive for LC3-II, The autophagosome then rapidly fuses with a lysosome to become a single-membrane autolysosome that co-stains for LC3-II and Lamp-1 and degrades the contents by acid hydrolases. Non infected cells contained little if any vesicles positive for LC3-II (Fig. 2C, left panel). In contrast, infection led to an overall increase in LC3 positive vesicles. Interestingly we observed that 31% of the bacteria co-localized within LC3-II positive vesicles whereas 45.7% of the bacteria were present in vesicles positives for LC3-II and Lamp-1 (Fig. 2C and Fig. S2B). We therefore determined whether AIEC survive within autolysosomes by affecting the lysosomal pH. For this purpose, uninfected and infected (3 h post infection) cells were labeled during the last 30 minutes of incubation with LysoTracker (green), a probe that detects acidic compartments. As expected, LysoTracker showed the presence of acidic compartments in uninfected neutrophil-like PLB 985 cells (Fig. 2D). However, following infection, all bacteria (blue staining for DNA) resided within non-acidic (LysoTracker negative) and LC3 positive vesicles.

The above results indicate that intracellular AIEC co-localized with the autophagy marker LC3 in infected neutrophil like cells. Invasive bacteria are targeted by host autophagy through three distinct pathways: *a*) autophagosomes may directly sequester bacteria free in the cytosol [26]; *b*) autophagosomes may engulf bacteria-containing phagosomes [27]; and *c*) LC3 may be recruited directly to bacteria-containing phagosomes thus stimulating phagosome maturation (LC3-associated phagocytosis [28]).

To investigate which of these pathways may be responsible for AIEC co-localization with LC3 in infected PBL cells, we studied the kinetics of infection and examined the cells with transmission electron microscopy (TEM). Thirty minutes after infection, 62% of the bacteria were localized within endosomes and 38% were present in canonical double-membrane autophagosomes (Fig. 2E and Fig. S2C). One hour after infection the percentage of bacteria within autophagosomes increased to 56.2% and fewer bacteria were present in endosomes (44.8%). Finally, after 2 h some bacteria (17.6%) were found to be free within the cytosol of infected cells. Interestingly, these bacteria were electron dense, suggesting that they were viable (see Fig. S2C). Together these results supported the hypothesis that AIEC can be targeted by canonical autophagy. Taken together, these findings established that the initial step of autophagic maturation (*i.e.* fusion of autophagosomes with lysosomes) occurred in AIEC-infected cells. However, the autolysosomes in which AIEC resided were non-degradative; indicating that the bacteria were capable of either delaying or preventing the full maturation of autolysosomes and of limiting the AIEC-induced inflammatory response.

### Upregulating Autophagy Kills Intracellular AIEC in Neutrophils and Limits the AIEC-Induced Inflammatory Response

**Figure 3:**
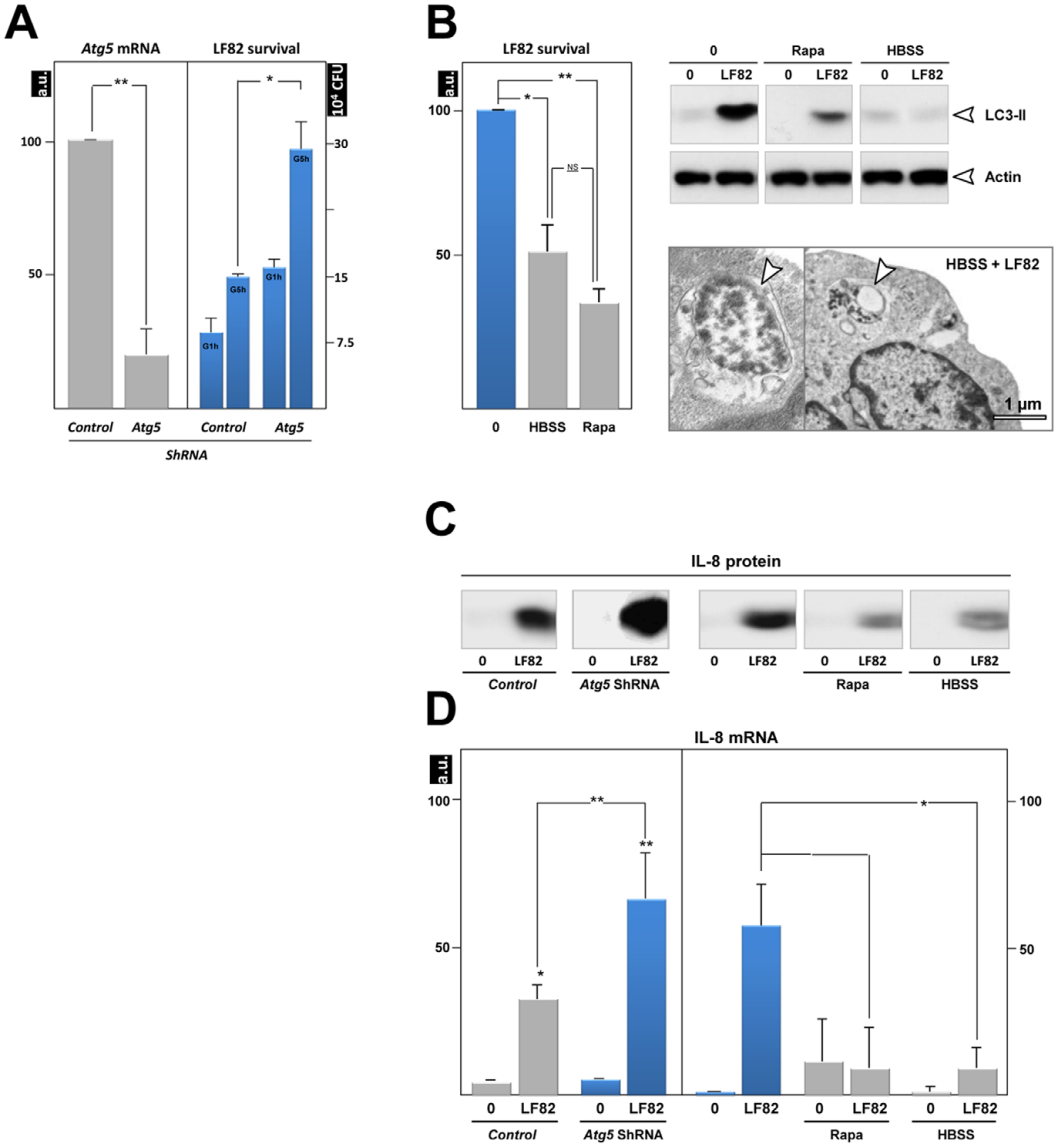
Modulation of autophagy affects AIEC survival. PLB-985 cells were transduced with either *Atg5* shRNA or the control shRNA, differentiated and then infected with LF82 (50 MOI). (**A**) A defect in autophagy (*Atg5* silencing) favours intracellular survival of bacteria. Intracellular bacteria were numbered 1 h and 5 h after addition of gentamycin. Data are means ± SEM of three experiments. *<0.01 and **p<0.003. Silencing of *Atg5* mRNA was confirmed by RT-PCR analysis (left). (**B**) Two inducers of autophagy, nutrient starvation (HBSS) and rapamycin (rapa, 100 nM in complete medium) decreased intracellular LF82 survival (left), rescued the autophagic flux (as shown by detection of LC3-II, upper right), and increased bacterial degradation (lower right, arrowhead, transmission electron micrograph). Modulation of LF82-induced IL-8 production in response to inhibition of autophagy (*Atg5* shRNA) or to stimulation of autophagy (rapamycin or starvation) was analyzed by immunoblotting (**C**) and qRT-PCR (**D**). The average ± S.D. is shown for three independent experiments, * <0.01 and ** p<0.003.

To gain insight into the role of autophagy in AIEC infection, we inhibited the formation of autophagosomes at the initiation step with *Atg5* short hairpin RNA (shRNA) in the neutrophil-like PLB-985 cells. The effective knockdown of *Atg5* was confirmed by qRT-PCR (Fig. 3A, left panel) and we verified that *atg5* silencing did not perturb cell differentiation and cell infection (see Fig. S3A to D). We found that AIEC survived intracellularly twice as much as in *Atg5*-depleted cells than in control cells (Fig. 3A, right panel). These results established that *Atg5*-dependent autophagy plays an essential role in controlling intracellular AIEC survival in differentiated neutrophils.

**Figure 4:**
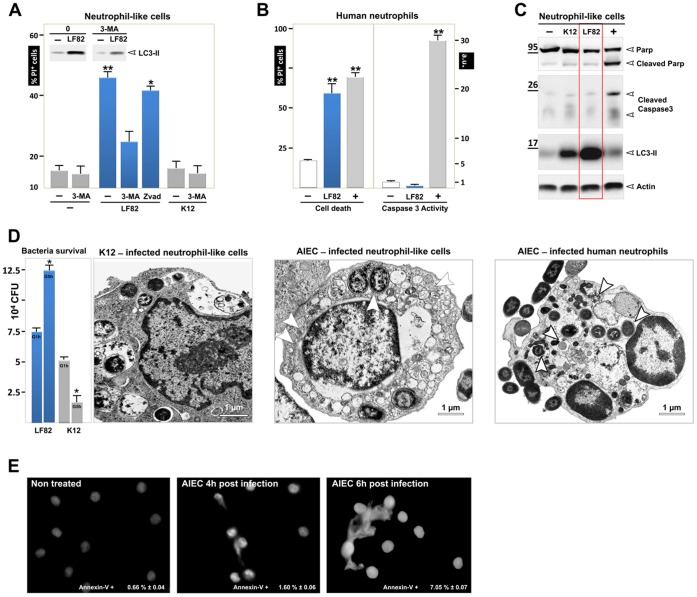
PMNs undergo autophagic death and NETosis on infection with AIEC LF82. Differentiated PLB-985 cells or PMN were infected (MOI 50) with K12 or AIEC LF82 bacteria for 1 h and gentamycin was added for the following 5 h, cells were then analysed. (**A**) Control or K12- or AIEC LF82-infected PLB-985 cells, treated or not with 3-methyladenine (3-MA, 5 mM, autophagy inhibitor) or Zvad (5 µM, a pancaspase inhibitor), were incubated with propidium iodide (PI) (5 µg/ml) and cell death was analysed by flow cytometric analysis as described in the materials and methods section. The average ± S.D. is shown for three independent experiments, *p<0.003. Inset: immunoblot analysis of LC3-II testifying to the induction of autophagy in infected cells. (**B,** left panel) PMNs were infected with AIEC LF82 and cell death was assayed as described in A. Maximal cell death (+) was obtained after treatment with etoposide phosphate (100 µg/ml). To test whether cell death was due to apoptosis, a caspase-3 activity test was performed as described in the materials and methods section (right panel). Maximal cell apoptosis (+) was obtained by treatment with staurosporine (10 µM). (**C**) Cleavage of PARP and caspase-3 in non-infected or K12- and AIEC LF82-infected differentiated PLB-985 cells were analysed by immunoblot analyses. Etoposide phosphate (100 µg/ml) was used as a positive control. LC3-II was detected in LF82-infected cells and compared to uninfected or K12-infected cells (1+5 h, MOI 50). Actin was used as a loading control. (**D**) Survival of AIEC LF82 and K12 in differentiated PLB-985 cells was analysed with the gentamycin assay (panel one). To compare the ultrastructural morphology of vesicles and bacteria in PLB-985 cells transmission electron microscopy was performed. Ultrastructural analysis of K12-infected differentiated PLB-985 cells shows bacterial sequestration and degradation in phagocytosis vacuoles (panel two). In contrast, accumulation of autophagic vesicles and bacteria (arrowheads) inside the vacuoles was observed in PLB-985 cells (panel three) and PMNs (panel four). Scale bar = 1 µm. (**E**) Differentiated PLB-985 were seeded on glass coverslips coated with poly D lysine (5 µg/cm^2^) and allowed to settle for 1 h. Cells were then infected with AIEC-LF82 (MOI 50) for 4 h (1 h+3 h) and 6 h (1 h+5 h), fixed with 3% paraformaldehyde and stained with Hoechst 33342 (0.5 µg/ml) to visualize DNA. Cells were examined with an epifluorescence Axiophot microscope (Zeiss). Early apoptosis and necrosis were assayed by measuring Annexin-V-fluos (Roche) and propidium iodide (PI). Differentiated non-infected PLB-985 cells or cells infected with AIEC LF82 (MOI 50) for 1 h+5 h were incubated with Annexin-V-fluos and PI and fluorescence was detected on a FACS Calibure.

In contrast, stimulation of autophagy by nutrient starvation (HBSS) or rapamycin treatment eliminated mycobacteria in macrophages [27], and AIEC in epithelial cells [6] and in macrophages [15]. We therefore addressed whether upregulating autophagy in neutrophils could reduce AIEC viability. Differentiated PLB cells were infected and incubated in nutrient deprived medium. Alternatively, following AIEC infection, cells were stimulated for 3 h with rapamycin, a pharmacological inducer of autophagy. Rapamycin treatment reduced by 70% the survival of AIEC in neutrophil-like cells (Fig. 3B, left panel). This anti-bacterial effect was due to the restoration of the autophagic flux, as shown by a decrease in the level of the substrate LC3-II of autophagy (Fig. 3B, right upper panels). As expected, physiological induction of autophagy by nutrient starvation (amino acid/serum-deprived HBSS medium) gave a similar decrease in the number of intracellular LF82 bacteria and restored the autophagic flux. Given that in macrophages autophagy helps to control the intensity of the inflammatory response [29], we next examined whether autophagy plays a similar role in AIEC-infected neutrophil-like cells. AIEC infection of PLB 985 cells dramatically induced the production of inflammatory cytokine IL-8 (Fig. 3C and D). Blocking autophagy with *Atg5* shRNA markedly increased IL-8 expression, while enhancing autophagy by starvation or rapamycin treatment had the opposite effect. The modulation of IL-8 production by autophagy was induced at the level of gene transcription and correlated with the intracellular bacterial load. Indeed, the non-pathogenic *E. coli* K12 strain, which does not survive within differentiated PLB-985 cells (see Fig. 4D), was a poor inducer of IL-8 mRNA expression (4-fold) whereas AIEC-LF82 bacteria induced a 25-fold increase (Fig. S3E). Therefore, in neutrophils, autophagy controls the production of inflammatory cytokines by eliminating pathogens.

### Infection with the *AIEC LF82* Strain Induced Autophagic Death of Neutrophils

At the site of infection, neutrophil viability is regulated and the neutrophil half-life is first enhanced to ensure the anti-microbial action. Once the invading bacteria are eradicated, neutrophils then undergo apoptosis to prevent tissue damage. Interestingly, many invasive pathogens (*Chlamydia pneumoniae*, *Pseudomonas aeruginosa* and *Staphylococcus aureus*) subvert neutrophils death (for review [30], [31]). Prompted by the observation that AIEC-infected macrophages do not undergo cell death [6], we examined whether AIEC infection can modulate neutrophils life span.

Neutrophil death was assessed by flow cytometry as measured by an increase in cell permeability to propidium iodide (PI). 15% of uninfected neutrophil-like PLB 985 cells were positive for propidium iodide. Infection with the AIEC LF82 strain gave rise to a 3.3-fold increase in the percentage of dying cells, which indicated that bacteria induced massive cell death in differentiated neutrophil-like cells (Fig. 4A). Similarly, AIEC LF82 infection induced death of human peripheral blood neutrophils (Fig. 4B).

Apoptosis, autophagy or necrosis trigger cell death. To distinguish which of these scenarios is triggering neutrophil death, we pretreated the cells with either 3-methyladenine (3-MA), which blocks autophagosome formation or Zvad, a well characterized inhibitor of caspase-dependent apoptosis. Interestingly, 3-MA treatment inhibited by 2-fold the percentage of PI positive cells under AIEC challenge, whereas Zvad had no effect. We then analyzed several biochemical and morphological hallmarks of apoptosis. Consistently, little, if any, caspase-3 enzyme activity was detected in AIEC-infected neutrophils, whereas the positive control increased by more than 30-fold the activity of the enzyme (Fig. 4B, right). Consistently, AIEC failed to induce cleavage of caspase-3 and of poly (ADP-ribose) polymerase (PARP) in differentiated neutrophil-like cells (Fig. 4C). Taken together, these data imply that AIEC LF82 induced a caspase-independent cell death in neutrophils.

Recently, excessive “autophagic” cell death (type II) has been reported to trigger non-apoptotic cell death in response to sustained stress [32]. Whether the ability of AIEC LF82 bacteria to survive within neutrophils produces a stress sufficient to induce autophagic cell death remains to be determined. To address this question, we studied the interaction of non-pathogenic bacteria with neutrophils. As shown in Figure 4A, infection with the non-pathogenic *E. coli* K12 strain had no effect on cell viability, suggesting that induction of neutrophil death is a feature of pathogenic bacteria. Interestingly, some bacteria invaded neutrophils, but their number decreased by more than 2-fold, indicating that they were killed within phagosomal/autophagic vesicles (Fig. 4D, left panel). In agreement with this hypothesis, K12 bacteria induced a lower level of LC3-II formation compared with LF82-infected cells (Fig. 4C) and we observed at the ultrastructural level that neutrophils infected with the non-pathogenic K12 bacteria did not contain autophagic vesicles (Fig. 4D). By contrast, we confirmed the induction of autophagic cell death by the accumulation of autophagic vesicles within the cytosol of both AIEC-infected neutrophil-like PLB-985 cells (Fig. 4D, third panel) and AIEC-infected human neutrophils (Fig. 4E, fourth panel). Meanwhile, the morphological hallmarks of apoptosis, such as cell shrinkage, membrane blebbing, nuclear condensation, or nuclear fragmentation, were not observed in these cells. Beside autophagy, activated neutrophils undergo an alternative cell death process called NETosis. NETosis consists of the release of neutrophil extracellular traps (NETs) composed of chromatin and granules of proteins, which bind and kill microorganisms [4]. Therefore we wondered whether AIEC-infected neutrophils could go into NETosis. Neutrophil trap formation was analyzed by immunofluorescence microscopy on coverslips seeded with differentiated PLB-985 cells infected or not with AIEC-LF82. As shown in Fig. 4E, bacterial infection resulted in the release of nuclear DNA into the extracellular medium; this phenomenon, clearly visible 6 h post infection, corresponded to NET formation. AIEC-induced NET formation is not linked to phosphatidylserine externalization since only 7% of 6 h post infected cells were annexin-V positive. Collectively, these data established that AIEC-infected neutrophils die as a result of both autophagic and NETosis cell death. However, a delay exists between these two cell death processes; autophagic cell death is visible 4 h post infection whereas NETosis is visible 6 h post infection. This suggests that autophagic cell death may be required to mediate NETosis.

## Discussion

Neutrophils are the most abundant cell type involved in the innate immune response. They are the first cells to be recruited to the site of infection where they engulf and kill invading microorganisms by classical phagocytosis. Indeed, we confirmed that the non-pathogenic *E. coli* K12 strain was phagocytosed and killed by the neutrophil-like PLB-985 cells (see Fig. 4). Beside phagocytosis, the autophagic pathway can play an important part in protecting host cells during bacterial infection, a process named xenophagy, and several pathogens have developed strategies to evade or even exploit this pathway [23]. Xenophagy-mediated killing of bacteria has so far been demonstrated in macrophages [27], [33], fibroblasts [34], [35], and epithelial cells [26], but no information is available concerning this process in neutrophils.

Using freshly isolated human neutrophils and the human neutrophil-like PLB-985 cells differentiated into mature neutrophils, we reported here that: *i*) neutrophils activated the autophagic machinery as an immediate response to fight AIEC LF82 infection and also to limit the production of inflammatory cytokines. Within the first 30 min of infection, AIEC were found within endosomes (vesicles free of intracellular components) and autophagic vesicles characterized by a double-membrane. These findings showed that AIEC LF82 bacteria entered cells via an endosomal compartment and were further captured by double-membrane autophagosomes. This indicates that AIEC LF82 bacteria are subjected to canonical autophagy instead of the recently described LC3-associated phagocytosis [28]; *ii*) Pathogenic AIEC evaded killing by neutrophil-like PLB cells by disturbing autophagic flux at the autolysosomal degradation step; *iii*) Rather than controlling infection, neutrophils died by AIEC-induced autophagic cell death, *iv*) Stimulation of cytoprotective autophagy by nutrient starvation or rapamycin treatment effectively overcame the AIEC-imposed block in autophagy and thereby reverted the ability of neutrophils to kill AIEC.

Neutrophil death is a key event in fighting infection. Indeed, it is well established that blood neutrophils are short-lived cells, programmed to die by apoptotic cell death [36]. However, several lines of evidence challenge this dogma. At an inflammatory site, a number of cytokines and bacterial products block neutrophils apoptosis to fight bacterial infection. In such an inflammatory environment, Simon HU *et al.* reported recently non-apoptotic neutrophil death that was related to massive PI3K-dependent autophagic vacuolization [3], [5].

In the present study we provided evidence of caspase-independent and PI3K-dependent cell death in bacteria-infected neutrophils. At the ultrastructural level, the hallmark of AIEC-infected cells was massive autophagic vacuolization, both in freshly isolated neutrophils and in the neutrophil-like PLB-985 cells. In contrast, dying neutrophils were not characterized by apoptotic features such as nuclei condensation/fragmentation and cell shrinkage. Likewise, the plasma membrane of AIEC-infected neutrophils was intact, excluding classical necrotic cell death. Of interest, this cell death was associated with autophagic (massive production of LC3II, and autophagic vacuolation) but not apoptotic markers (caspase 3 activity, caspase 3 cleavage, and Parp cleavage). In line with this observation, AIEC-infected cells were rescued by a pharmacological inhibitor of autophagy but not of apoptosis. Taken together, AIEC-infected neutrophils underwent autophagic cell death.

A similar caspase-independent death pathway has been described *in vitro* in neutrophils stimulated with inflammatory mediators such as TNF-α, Siglec-9, CD44 ligation and GM-CSF [3], [5], [37]. Interestingly, neutrophil vacuolization has been observed in patients with inflammatory diseases, such as rheumatoid arthritis and septis, which demonstrates that this type of cell death occurs *in vivo* [38].

A few years ago the group of Zychlinsky provided evidence of a novel form of neutrophil cell death process called NETosis [39]. Neutrophil extracellular traps (NETs), which are made of chromatin and granules of proteins, allow efficient killing of microorganisms. We demonstrated in the present study that AIEC-infected neutrophils underwent NETosis. Interestingly, this process takes place after autophagy since it requires 6 h of infection whereas autophagy is visible at 1 h post infection with AIEC and reached a plateau by 3 h. Therefore, we characterized the link between autophagy and NETosis by studying trap formation in autophagy deficient PLB Sh-*atg5* cells. As shown in Fig. S4 differentiated PLB Sh-*atg5* cells infected with AIEC-LF82 (MOI 50) for 6 h (1 h+5 h) or treated with 25 nM PMA (as a positive control) were unable to form Traps. This finding was confirmed by two independent studies. In the first one, Remijsen and co-authors demonstrated that PMA-induced NETosis, a mechanism dependent on autophagy and ROS production, was blocked when autophagy is inhibited [40]. In the second, Mitroulis and co-authors have shown that NET production observed in neutrophils from patient with acute gout was inhibited when autophagy was prevented [41]. All together, these findings suggest that autophagic cell death may commit cells to NETosis.

The autophagic neutrophil death that we observed on pathogenic bacterial infection may be of physiological importance. Indeed, the non-pathogenic *E. coli* K12 strain was phagocytized, did not affect cell viability, did not induce IL-8 and was efficiently killed by neutrophils. In contrast, invading AIEC LF82 bacteria induced production of IL-8 and subverted the autophagy pathway as a safe replicative niche. At a later time, AIEC LF82 bacteria overload caused accumulation of autophagic vesicles and cell death. The first obvious effect of this particular cell death was that the plasma membrane of dying neutrophils remained intact, and therefore should not induce further *in situ* inflammation. The second benefit of this cellular suicide is the killing of intracellular AIEC, as suggested by the decrease in intracellular CFU at late times of infection. Similarly, other bacterial pathogens (*Clostridium difficile*, *Legionella pneumophila*, *Salmonella enterica* serovar *typhimurium*, and *Vibrio cholera*, *Staphylococcus aureus*) that are armed with pili and flagella have been described to subvert the autophagic machinery not only to multiply but also to kill the host epithelial cell by a caspase-independent pathway [42], [43].

It should be noted that AIEC induced death in PMNs, but not in macrophages [8] or epithelial cells [6]. This suggests that, within the intestinal epithelium, PMNs have a unique sensitivity to autophagic cell death upon AIEC infection. Together with apoptosis, we therefore propose that autophagic cell death may be a safeguard mechanism to control the number of PMN and thereby limit PMN-mediated chronic inflammation. This mechanism may play a critical role under pathologic conditions. Indeed, a defect in xenophagy due to Atg16L and IRGM polymorphisms has recently been implicated in the pathogenesis of CD [37]. We used freshly isolated PMNs from healthy donors and we checked that the PLB-985 cell line was wild-type for Atg16L and IRGM autophagic genes. Future work may answer the important issue of whether impairment of the initiation of autophagy by Atg16L and IRGM polymorphisms in CD patients can impair xenophagy and at the same time preserve neutrophil function, and thereby promote prolonged, tissue-damaging chronic inflammation. Understanding the mechanisms involved in PMN xenophagic/autophagic cell death may be of essential interest in developing novel pharmacological therapies to control inflammatory diseases.

## Supporting Information

Figure S1 **Characterization of differentiated PLB-985.** A) Granulocytic differentiation was checked by morphological analysis of cytocentrifuged cells stained with May-Grunwald-Giemsa solution. Neutrophilic maturation is indicated by an increase in pyknosis of nuclei, loss of nucleoli and the appearance of azurophilic granularity in the cytoplasm. B) CD11b expression at the cell surface of non-differentiated PLB-985 cells or cells differentiated for 6 days was analyzed by flow cytometry. Control isotypic (dashed line) superposed with the profile of non-differentiated cells (blue line) whereas the profile of differentiated PLB985 (pink line) migrated to the right. Under this condition we observed a highly reproducible 3-fold increase in CD66b expression (n = 3). C) Transmission electron microcopy of non-differentiated or differentiated PLB-985 cells indicated a reduction in nuclei, nuclear indentation and an increase in electron-dense granules in the cytoplasm (arrow heads). D) Stable expression of CD11b by differentiated PLB-985 cells. A time course of expression of CD11b, measured by flow cytometry, of differentiated or non-differentiated cells demonstrated stable expression of CD11b by differentiated PLB-985 cells.
(TIF)

Figure S2 **Co-localization of AIEC LF82 bacteria with LC3-II and Lamp-1 in differentiated PLB-985 cells.** A) Representative confocal images of cells infected with AIEC-LF82-GFP bacteria (1+3 h post infection) showed the co-localization of bacteria with LC3-II and Lamp-1 as indicated by white punctiform staining (white arrow head). We show, in the two insets, extranuclear small dots stained with Dapi that correspond to bacteria, all small blue dots are GFP positive. Bar = 5 µm B) Quantification of co-localization of bacteria with LC3-II, Lamp-1 and lysotracker. Pictures of 30 bacteria were taken at x 63 magnification and three-dimensional images were used to assess the co-localization of bacteria (blue) with autophagosomes (green) and autolysosomes (green and red) or bacteria with acidic LC3-II positive vesicles (green and red). Experiments were done in triplicate and quantification is expressed as the mean of 3 experiments in which 30 bacteria were counted per experiment. C) Representative TEM images showing bacteria within endosomes that correspond to single membrane vesicles free of intracellular components (left upper panel, Bar = 200 nm) and double membrane autophagosomes (inset Bar = 200 nm) containing bacteria and cytoplasmic components (left lower panel, Bar = 1 µm) in LF82-infected neutrophils (1 h post infection). Right panel: quantification of bacteria within intracellular organelles. Pictures of 20 bacteria at 200 nm resolution were taken to perform statistical analysis.
(TIF)

Figure S3 **Characterization of PLB sh-** ***atg5*** **cells.** A) Granulocytic differentiation was checked by morphological analysis of cytocentrifuged cells stained with May-Grunwald-Giemsa solution. B) CD11b expression at the cell surface of non-differentiated PLB Sh-*atg5* (green line) or cells differentiated for 6 days (purple profile) was analyzed by flow cytometry. C) AIEC LF82 bacteria induced autophagy in both PLB Sh-ctr and PLB Sh-*atg5* cells. The level of LC3-II protein expression was analyzed by immunoblotting of cells infected with bacteria at a MOI of 50. Actin staining was used as a control for protein loading. D) Survival of AIEC-LF82 bacteria in differentiated PLB Sh-ctr and Sh-*atg5* cells. Cells were infected (1 h+3 h) with AIEC-LF82 bacteria fixed with paraformaldehyde and stained with DAPI (blue) and actin (red). The number of live intracellular bacteria (small intracellular blue dots) is increased in PLB Sh-atg5 cells versus Sh-ctr cells. Bar = 5 µm. E) Expression of IL-8 by differentiated PLB-985 cells in response to *E. coli* K12. IL-8 levels (mRNA, left panel and protein, right panel) were studied in response to non-pathogenic *E. coli* K12 strain versus AIEC LF82 infection.
(TIF)

Figure S4 **No NETosis in differentiated PLB Sh-** ***atg5*** **cells infected with AIEC-LF82 bacteria.** Differentiated PLBSh-*atg5* were seeded on glass coverslips coated with poly D lysine (5 µg/cm^2^) and allowed to settle for 1 h. Cells were then infected with AIEC-LF82 (MOI 50) for 4 h (1 h+5 h) or treated with 25 nM PMA, fixed with 3% paraformaldehyde and stained with Hoechst 33342 (0.5 µg/ml). Cells were examined with an epifluorescence Axiophot microscope (Zeiss). Tables, showed early apoptosis and necrosis, assayed by measuring Annexin-V-fluos (Roche) and propidium iodide (PI). Differentiated non-infected PLB Sh-*atg5* cells, cells infected with AIEC LF82 (MOI 50) for 1 h+5 h or cells treated with anisomycin (10 µg/ml, positive control) were incubated with Annexin-V-fluos and PI as recommended by the manufacturer. Fluorescence was examined on a FACS Calibure. This experiment showed that differentiated PLB Sh-*atg5* cells were unable to go into NETosis. We also observed nuclear condensation in each of the conditions tested, which suggested that cells impaired in autophagy underwent apoptosis.
(TIF)

Table S1 **Sequence of the Primers used in this study.**
(PDF)
