# Supplementary figures and images for: Subversion of Autophagy in Adherent Invasive Escherichia coli-Infected Neutrophils Induces Inflammation and Cell Death
Source: PLoS One. 2012 Dec 14;7(12):e51727. doi: 10.1371/journal.pone.0051727 (PMC3522719; doi:10.1371/journal.pone.0051727)

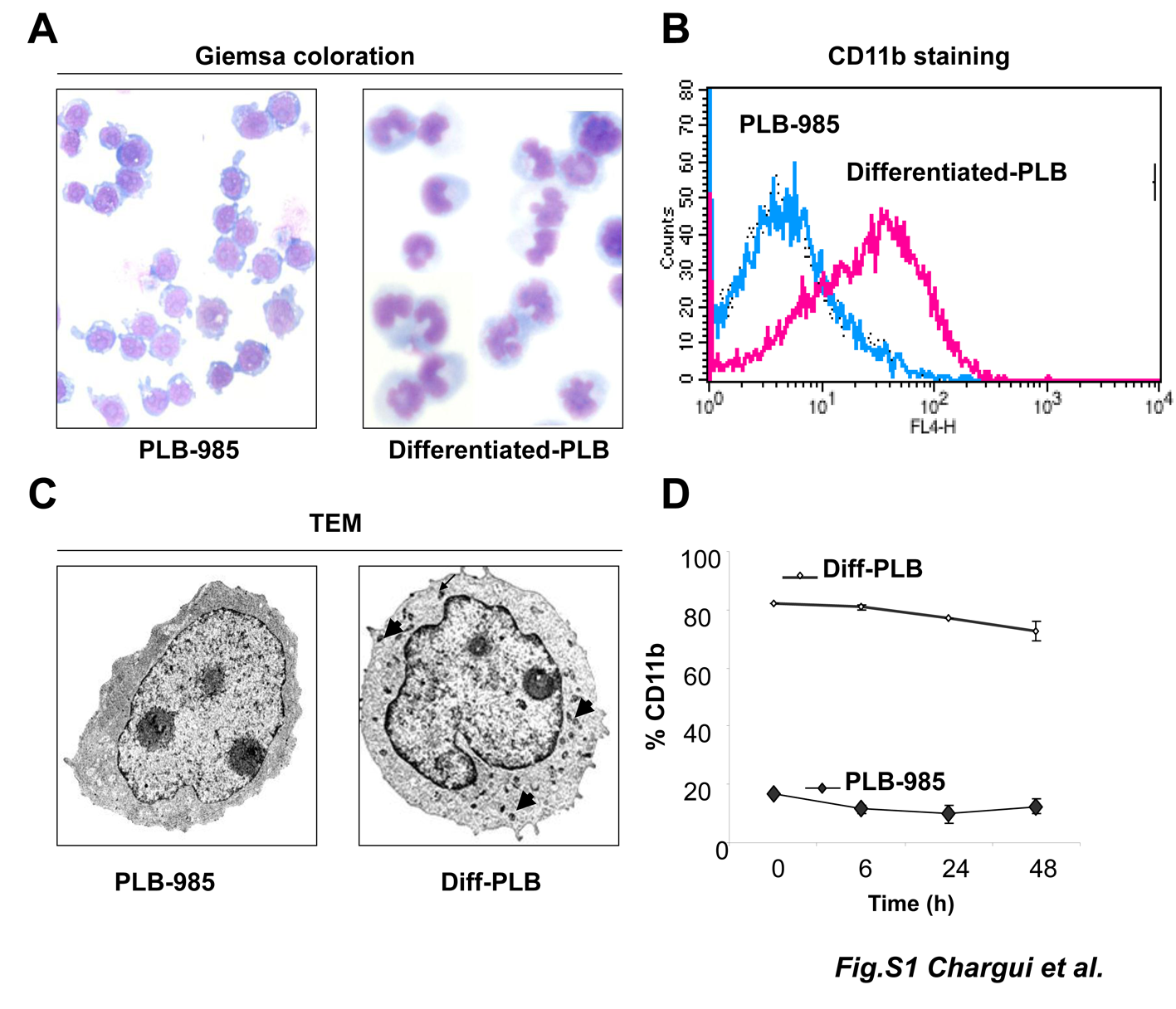

Supplement: Figure S1 — Characterization of differentiated PLB-985. A) Granulocytic differentiation was checked by morphological analysis of cytocentrifuged cells stained with May-Grunwald-Giemsa solution. Neutrophilic maturation is indicated by an increase in pyknosis of nuclei, loss of nucleoli and the appearance of azurophilic granularity in the cytoplasm. B) CD11b expression at the cell surface of non-differentiated PLB-985 cells or cells differentiated for 6 days was analyzed by flow cytometry. Control isotypic (dashed line) superposed with the profile of non-differentiated cells (blue line) whereas the profile of differentiated PLB985 (pink line) migrated to the right. Under this condition we observed a highly reproducible 3-fold increase in CD66b expression (n = 3). C) Transmission electron microcopy of non-differentiated or differentiated PLB-985 cells indicated a reduction in nuclei, nuclear indentation and an increase in electron-dense granules in the cytoplasm (arrow heads). D) Stable expression of CD11b by differentiated PLB-985 cells. A time course of expression of CD11b, measured by flow cytometry, of differentiated or non-differentiated cells demonstrated stable expression of CD11b by differentiated PLB-985 cells. (TIF) [file pone.0051727.s001.tif]

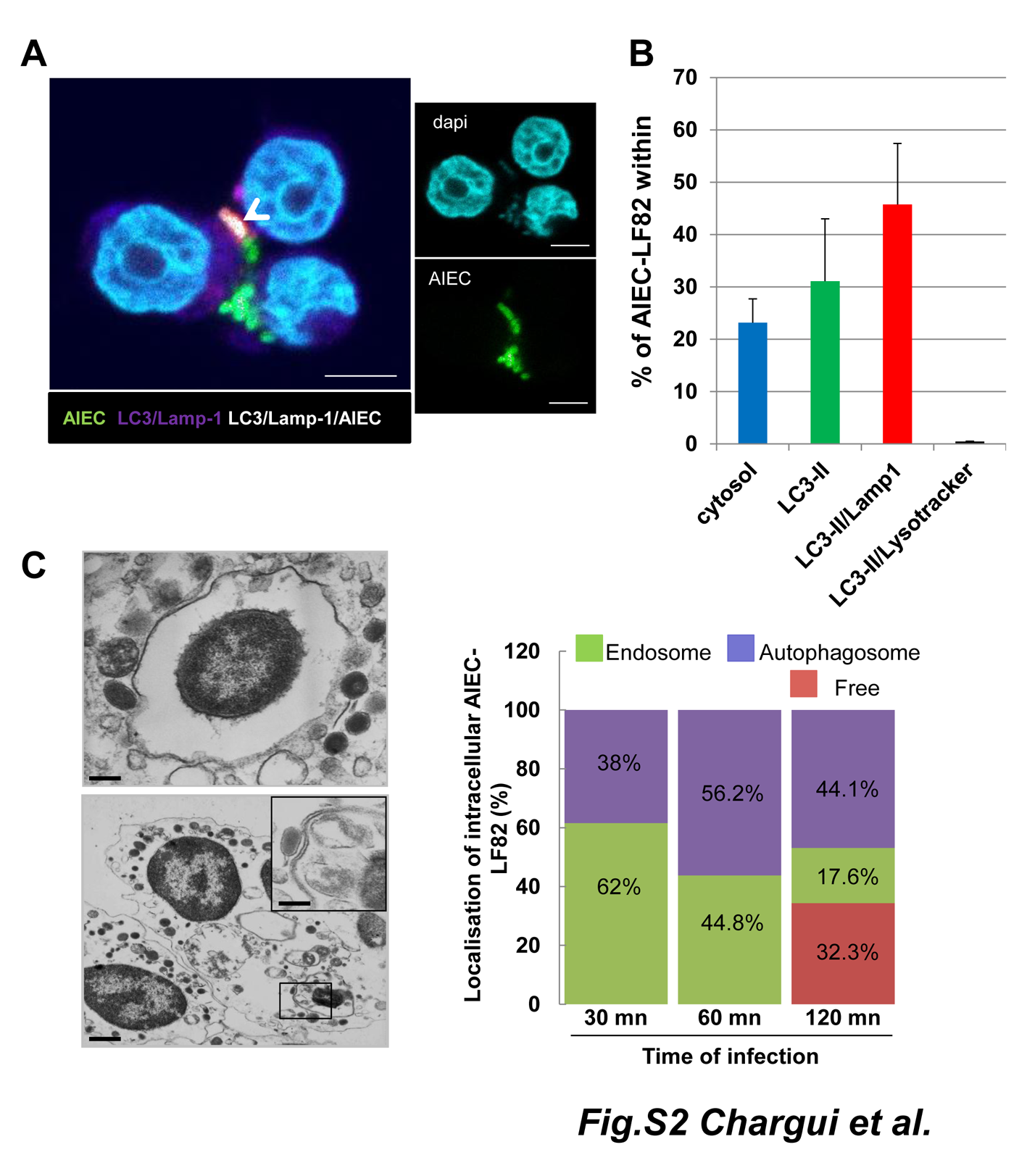

Supplement: Figure S2 — Co-localization of AIEC LF82 bacteria with LC3-II and Lamp-1 in differentiated PLB-985 cells. A) Representative confocal images of cells infected with AIEC-LF82-GFP bacteria (1+3 h post infection) showed the co-localization of bacteria with LC3-II and Lamp-1 as indicated by white punctiform staining (white arrow head). We show, in the two insets, extranuclear small dots stained with Dapi that correspond to bacteria, all small blue dots are GFP positive. Bar = 5 µm B) Quantification of co-localization of bacteria with LC3-II, Lamp-1 and lysotracker. Pictures of 30 bacteria were taken at x 63 magnification and three-dimensional images were used to assess the co-localization of bacteria (blue) with autophagosomes (green) and autolysosomes (green and red) or bacteria with acidic LC3-II positive vesicles (green and red). Experiments were done in triplicate and quantification is expressed as the mean of 3 experiments in which 30 bacteria were counted per experiment. C) Representative TEM images showing bacteria within endosomes that correspond to single membrane vesicles free of intracellular components (left upper panel, Bar = 200 nm) and double membrane autophagosomes (inset Bar = 200 nm) containing bacteria and cytoplasmic components (left lower panel, Bar = 1 µm) in LF82-infected neutrophils (1 h post infection). Right panel: quantification of bacteria within intracellular organelles. Pictures of 20 bacteria at 200 nm resolution were taken to perform statistical analysis. (TIF) [file pone.0051727.s002.tif]

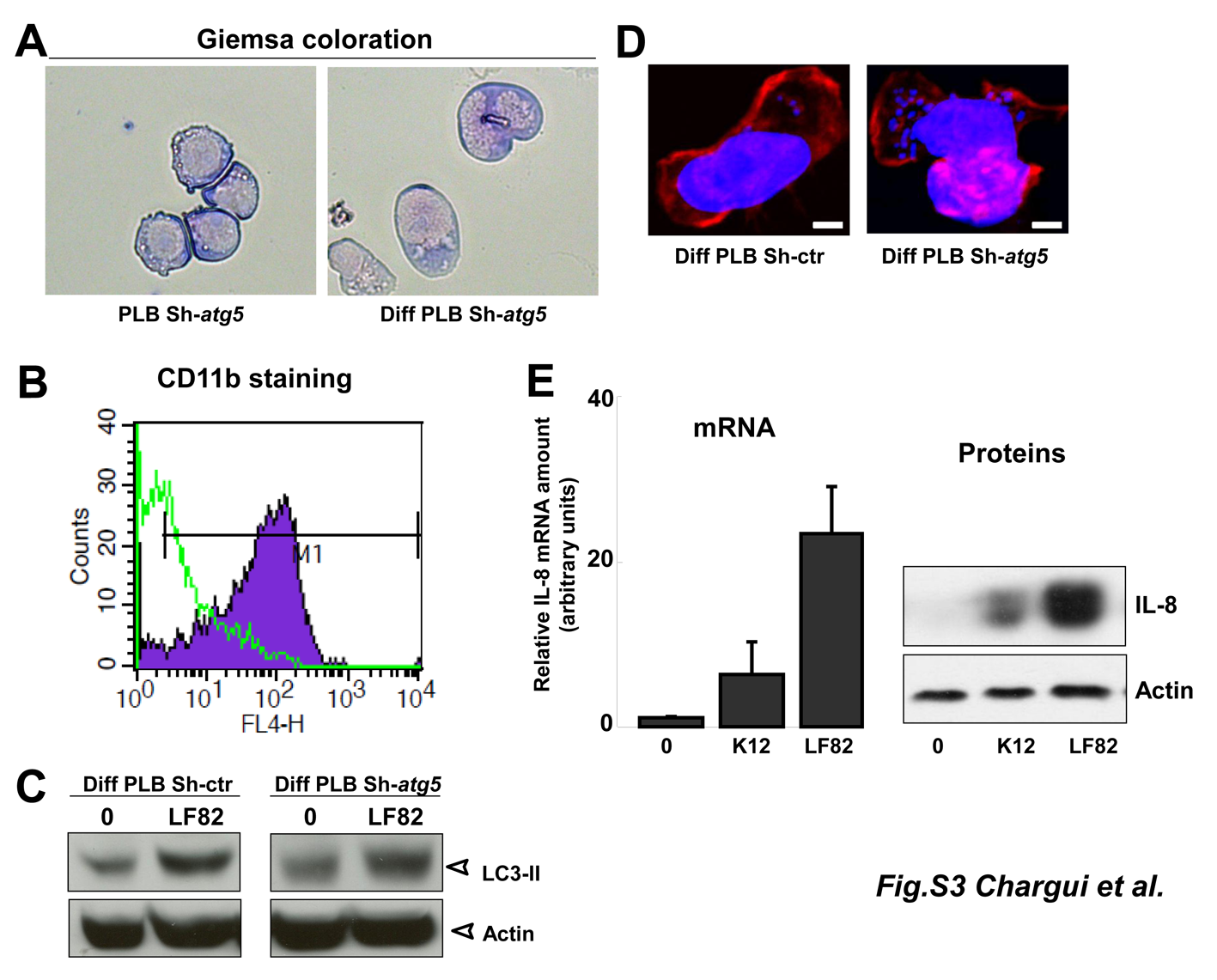

Supplement: Figure S3 — Characterization of PLB sh- atg5 cells. A) Granulocytic differentiation was checked by morphological analysis of cytocentrifuged cells stained with May-Grunwald-Giemsa solution. B) CD11b expression at the cell surface of non-differentiated PLB Sh-atg5 (green line) or cells differentiated for 6 days (purple profile) was analyzed by flow cytometry. C) AIEC LF82 bacteria induced autophagy in both PLB Sh-ctr and PLB Sh-atg5 cells. The level of LC3-II protein expression was analyzed by immunoblotting of cells infected with bacteria at a MOI of 50. Actin staining was used as a control for protein loading. D) Survival of AIEC-LF82 bacteria in differentiated PLB Sh-ctr and Sh-atg5 cells. Cells were infected (1 h+3 h) with AIEC-LF82 bacteria fixed with paraformaldehyde and stained with DAPI (blue) and actin (red). The number of live intracellular bacteria (small intracellular blue dots) is increased in PLB Sh-atg5 cells versus Sh-ctr cells. Bar = 5 µm. E) Expression of IL-8 by differentiated PLB-985 cells in response to E. coli K12. IL-8 levels (mRNA, left panel and protein, right panel) were studied in response to non-pathogenic E. coli K12 strain versus AIEC LF82 infection. (TIF) [file pone.0051727.s003.tif]

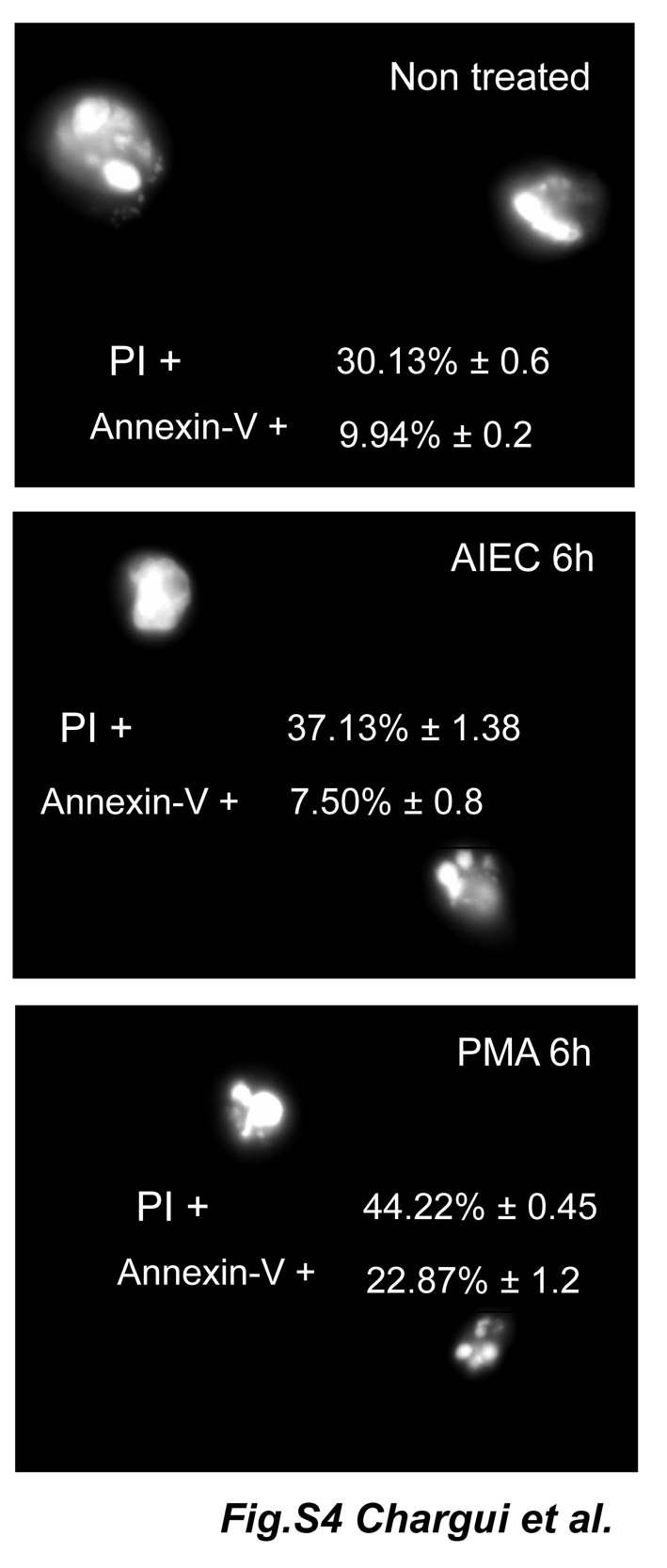

Supplement: Figure S4 — No NETosis in differentiated PLB Sh- atg5 cells infected with AIEC-LF82 bacteria. Differentiated PLBSh-atg5 were seeded on glass coverslips coated with poly D lysine (5 µg/cm2) and allowed to settle for 1 h. Cells were then infected with AIEC-LF82 (MOI 50) for 4 h (1 h+5 h) or treated with 25 nM PMA, fixed with 3% paraformaldehyde and stained with Hoechst 33342 (0.5 µg/ml). Cells were examined with an epifluorescence Axiophot microscope (Zeiss). Tables, showed early apoptosis and necrosis, assayed by measuring Annexin-V-fluos (Roche) and propidium iodide (PI). Differentiated non-infected PLB Sh-atg5 cells, cells infected with AIEC LF82 (MOI 50) for 1 h+5 h or cells treated with anisomycin (10 µg/ml, positive control) were incubated with Annexin-V-fluos and PI as recommended by the manufacturer. Fluorescence was examined on a FACS Calibure. This experiment showed that differentiated PLB Sh-atg5 cells were unable to go into NETosis. We also observed nuclear condensation in each of the conditions tested, which suggested that cells impaired in autophagy underwent apoptosis. (TIF) [file pone.0051727.s004.tif]
