# Supplementary material for: Subversion of Autophagy in Adherent Invasive Escherichia coli-Infected Neutrophils Induces Inflammation and Cell Death
Source: PLoS One. 2012 Dec 14;7(12):e51727. doi: 10.1371/journal.pone.0051727 (PMC3522719; doi:10.1371/journal.pone.0051727)
Supplement: Table S1 — Sequence of the Primers used in this study. (PDF) [file pone.0051727.s005.pdf]

Table S1: Primer sequences used in this study:

il-8: 5′–ATGACTTCCAAGCTGGCCGTG–3′ (forward)

5′–CTTGAG AGTGGCATAGACTTCTGTCT–3′ (reverse);

***atg5*:** 5′-GCCATAGCTTGGAGTAGGTTTG GC–3′ (forward)

5′–GCGTGAAACAAGTTGGAATTCGTCC–3′ (reverse);

p62: 5′–CT TCCTGACGCTGCTGCTCTTCTGC–3′ (forward)

5′–GCTCAGCTGCGTGCAACCG ATC–3′ (reverse),

36B4: 5'–TCCAGGCTTTGGGCATCA–3' (forward)

5'–TCTCAGCC CTCTTCAAAAACT–3' (reverse)
